# IUC24418-84 Biomarkers detecting minimal residual disease for predicting risk of relapse in operable urothelial cancer: a systematic review and meta-analysis of phase II/III clinical trials

**DOI:** 10.1093/oncolo/oyaf248.010

**Published:** 2025-08-29

**Authors:** A C Redla, S Mehrotra, J K Tan, A Ghose, A Shah, P Kotecha, A Sharma, P Das, J Teoh, N Vasdev

**Affiliations:** King’s College London, London, United Kingdom; King’s College London, London, United Kingdom; University of Manchester, Manchester, United Kingdom; Barts Cancer Centre, London, United Kingdom; University College London, London, United Kingdom; Barts Cancer Institute, Queen Mary University of London, London, United Kingdom; Mount Vernon Cancer Centre, Northwood, United Kingdom; University Hospitals of Derby and Burton NHS Foundation Trust, Derby, United Kingdom; The Chinese University of Hong Kong, Hong Kong, China; University of Hertfordshire, Hatfield and East and North Hertfordshire Teaching NHS Trust, Stevenage, United Kingdom

## Abstract

**Background:**

Non-metastatic urothelial cancers (UC), whether non-muscle-invasive bladder cancer (NMIBC) (T_is_/T_a_/T_1_) or MIBC (T_2_/T_3_/T_4_), are known for high recurrence and variable treatment response, henceforth warranting reliable biomarkers to guide therapy and surveillance. Circulating tumor DNA (ctDNA) and urinary assays show promise, but their clinical utility remains uncertain. Our objective was to systematically review and evaluate biomarkers detecting minimal residual disease (MRD) post-surgery for predicting the risk of relapse.

**Methods:**

Our systematic review included a MEDLINE database search for phase II/III clinical trials implementing blood or urine biomarkers detecting MRD (intervention) vs standard of care, that is, without biomarkers (comparator) in adult patients with operable UC (population). Out of 1,667 records from 2006 to June 2025, 16 underwent a full text screen, with 2 meeting inclusion criteria. A meta-analysis using RStudio assessed the association between biomarker positivity and disease recurrence.

**Results:**

Two clinical trials—a single-center parallel-arm, phase II (PMID 39406613, NCT01687244) on NMIBC and a multi-center randomized controlled phase III (PMID 34135506, NCT02450331) on MIBC were included. Out of 598 pooled evaluable patients, 581 underwent blood-based circulating tumor DNA (ctDNA) testing, whereas 18 had urine-based urinary tumor DNA (utDNA) profiling. Biomarker positivity was associated with MRD presence. A random-effects meta-analysis combining both trials yielded a pooled hazard ratio (HR) of 6.19 (95% CI: 4.42-8.69) for disease recurrence in biomarker-positive vs biomarker-negative patients. This indicates a significantly increased risk of relapse associated with MRD positivity. On individual analysis, ctDNA was strongly associated with disease recurrence (HR: 6.30; 95% CI, 4.45-8.92; *P* < .0001), whereas utDNA also showed significant association, albeit with higher uncertainty (HR: 4.60; 95% CI, 1.07-19.77; *P* = .04).

**Conclusions:**

Our findings suggest that blood-based ctDNA and urine-based utDNA are effective markers of MRD in operable UC. Both were predictive of the risk of relapse, although ctDNA demonstrated relatively more precise prognostic performance than utDNA. Incorporating these biomarkers into clinical surveillance protocols may enable early detection of relapse and guide post-treatment monitoring.

